# Reversible Dilated Cardiomyopathy in a Male Patient With Rheumatoid Arthritis: A Case Report

**DOI:** 10.7759/cureus.72216

**Published:** 2024-10-23

**Authors:** Cristina M Padovani, Jennifer Tao, Mohammad I Fardos, Linda Brecher

**Affiliations:** 1 Rheumatology, Rowan-Virtua School of Osteopathic Medicine, Stratford, USA; 2 Internal Medicine, Jefferson Health, Stratford, USA; 3 Dermatology, HCA Florida Largo Hospital, Largo, USA; 4 Dermatology, Rowan-Virtua School of Osteopathic Medicine, Stratford, USA; 5 Rheumatology, Orlando College of Osteopathic Medicine, Orlando, USA

**Keywords:** arthritis and orthopedic rheumatology, dilated cardiomyopathy (dcm), hydroxychloroquine, rheumatoid arthritis, rituximab therapy

## Abstract

This case describes a rare instance of reversible dilated cardiomyopathy (DCM) in a 65-year-old Caucasian male with a significant past medical history of inflammatory rheumatoid arthritis (RA) controlled with rituximab and hydroxychloroquine (HCQ). The patient presented with acute onset of dyspnea on exertion and palpitations and was diagnosed with congestive heart failure in the context of DCM. Despite having no prior cardiac abnormalities, an EKG revealed a new left bundle branch block, and an echocardiogram demonstrated a severely reduced left ventricular ejection fraction (LVEF) of 10-15%. Left heart catheterization and coronary angiography revealed no evidence of coronary artery disease. Given the absence of an overt cause, drug-induced DCM was suspected; hence, rituximab and HCQ were discontinued. Other common causes of DCM, including alcohol abuse and virus-induced DCM, were excluded based on relevant testing. Seven to nine months after cessation of HCQ and rituximab, the patient’s RA progressed, and treatment was initiated with IV tocilizumab, resulting in a good clinical response. At the 26-month follow-up, a repeat echocardiogram revealed mild mitral regurgitation with an LVEF which improved to 55%. At this point, Takotsubo cardiomyopathy was considered a potential cause of this patient’s DCM due to its reversible nature. This case highlights the importance of comprehensive cardiac monitoring in symptomatic patients at high risk for cardiovascular disease, such as this patient with long-standing inflammatory disease. Physicians should work together to closely monitor and consider the serious potential risks of all treatment regimens.

## Introduction

Cardiomyopathy (CM) is broadly classified as a group of diseases that affect the myocardium, resulting in abnormal thickening, stiffening, or thinning of the heart muscle [1]. The heart’s ability to pump blood around the body is reduced, and its ability to maintain a normal electrical rhythm is negatively affected [1]. As CM progresses, arrhythmias and congestive heart failure (CHF) can occur. Dilated cardiomyopathy (DCM) is the most common type of CM worldwide, with a prevalence of 1:2,500, and there is an estimated incidence of 7 cases per 100,000 people per year [2-5]. DCM is characterized by left ventricular (LV) systolic dysfunction, with an associated increase in both mass and volume [3]. Clinical manifestations include shortness of breath, fatigue, exercise intolerance, abdominal pain, pallor, and swelling. There are various etiologies associated with DCM which include ischemic heart disease, infections, medications, and idiopathic causes. Common therapeutics implicated in the development of DCM include antineoplastic medications such as anthracyclines and trastuzumab [3].

Rituximab is a monoclonal antibody that binds to the CD20 antigen found on the surface of B lymphocytes. It is commonly used in the treatment of white blood cell cancers such as non-Hodgkin’s lymphoma but has also been used to treat autoimmune disorders such as rheumatoid arthritis (RA) and vasculitis [6]. Although there are rare cases of reversible drug-induced cardiotoxicity reported in the literature [2,4,6], this condition is generally understood to be irreversible, as likely mechanisms of action include cardiomyocyte necrosis and apoptosis. Direct damage to the cardiomyocytes leads to stretching, which is not reversible [7]. Further, these reversible instances of rituximab-induced DCM in the literature primarily occurred during the infusion of the biologic therapy or relatively soon after rituximab infusion (<6 months) [2,4].

Hydroxychloroquine (HCQ) is an antimalarial drug that was originally developed for the treatment of *Plasmodium falciparum* infections but is now also widely used for the treatment of many rheumatic diseases due to its safety, cost, and effectiveness. HCQ’s efficacy in the treatment of rheumatic diseases such as RA and systemic lupus erythematosus can be attributed to its anti-inflammatory and immunomodulatory properties [8]. HCQ has been occasionally implicated in cases of myopathy; some studies suggest that this is due to prolonged use of HCQ, as this can lead to the development of cardiac conduction disturbances, such as bundle branch blocks (BBBs) and atrioventricular (AV) blocks, as well as in CM [9-11]. These conduction disturbances can eventually lead to the development of cardiomyocyte stretching and subsequent dilation and CM [7]. Despite these reported instances of HCQ-induced cardiotoxicity, cardiac side effects are rarely reported by patients undergoing HCQ therapy.

## Case presentation

This case describes a rare instance of reversible DCM in a male patient with RA. A 65-year-old Caucasian male with a significant past medical history of well-controlled inflammatory RA for 30 years presented with the acute onset of dyspnea on exertion and palpitations and was diagnosed with CHF in the context of DCM. Before this presentation, the patient had stable RA treated with oral methotrexate 20 mg/week and folic acid 1 mg/day for six years. This RA treatment was successful at achieving low disease activity for the patient, and the patient did very well until he developed non-Hodgkin’s lymphoma (NHL; diffuse large B-cell lymphoma).

As a result of the patient’s NHL, methotrexate was discontinued, and the patient was treated with rituximab, cyclophosphamide, doxorubicin, vincristine, and prednisone (R-CHOP). During and after lymphoma treatment, the patient had a complete remission of his RA. After completion of chemotherapy, his RA remained in remission until about one year later when he experienced a progressive increase in joint pain, synovitis, and stiffness. Treatment for recurrence of RA was then initiated with rituximab and HCQ. The patient again achieved remission of RA.

Rituximab and HCQ were continued until the patient presented after seven years of treatment with the acute onset of dyspnea on exertion and palpitations. He was hospitalized and evaluated for CHF. The patient had no known prior cardiac abnormalities or defects as per the patient himself and his provided chart. EKG revealed a new left bundle branch block (LBBB) and echocardiogram revealed his left ejection fraction (LVEF) to be 10-15% (normal LVEF = 55-75%), with moderate-to-severe mitral regurgitation and global cardiac hypokinesis.

The patient was diagnosed with CHF in the context of DCM, with the cause unknown at the time. Common causes of DCM were considered and ruled out. The patient’s medical history, present history, and current medical status did not reveal signs or symptoms of alcohol use disorder. Laboratory testing for the typical viral antibodies associated with cardiomyopathy was negative. In our patient, cardiac catheterization revealed no evidence of coronary artery disease, and cardiac MRI revealed an enlarged left ventricle with global hypokinesis. The patient also underwent left heart catheterization, left coronary angiography, and right coronary angiography. The results revealed no evidence of coronary artery disease, and these tests only showed minor luminal irregularities. A cardiac MRI was subsequently completed and revealed an enlarged LV of normal thickness but with global cardiac hypokinesis.

In-patient treatment for heart failure was started and he was discharged on furosemide 40 mg/day, metoprolol XL 50 mg/day, and lisinopril 10 mg/day. A life vest was also prescribed. His advised activity level included activities of daily living and no strenuous exercise. The patient was advised to proceed with the implantation of a biventricular implantable cardioverter-defibrillator (ICD). The patient was hesitant to move forward with surgery for ICD, as he was hopeful that his cardiac function would improve over time, but he ultimately did undergo ICD placement.

Over the next several months, the patient was closely followed by cardiology and rheumatology. He was referred to cardiac rehabilitation; his cardiac status remained stable, and he had no worsening symptoms of dyspnea or edema. As there was no overt cause of the patient’s DCM, drug-induced DCM was eventually considered; therefore, HCQ was discontinued, and rituximab infusions were not continued at that time. At this time, he was not on any disease-modifying antirheumatic drugs (DMARDs), and there was only a slight improvement in his LVEF to 23%.

**Figure 1:**
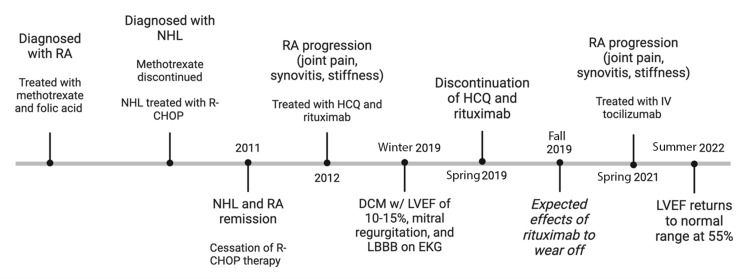
Case presentation timeline. RA: rheumatoid arthritis; NHL: non-Hodgkin’s lymphoma; R-CHOP: rituximab, cyclophosphamide, doxorubicin, vincristine, and prednisone; HCQ: hydroxychloroquine; DCM: dilated cardiomyopathy; LVEF: left ventricular ejection fraction; LBBB: left bundle branch block

Over the course of the next several months, he experienced progression of his RA with joint pain, synovitis of metacarpophalangeal, metatarsophalangeal, wrists, elbows, stiffness, and difficulty with his daily activities. At this time, treatment was initiated with IV tocilizumab, initially at 4 mg/kg every four weeks, and then increased to 8 mg/kg every four weeks. The patient’s clinical response was good with notable improvement in his stiffness, synovitis, and function. On follow-up 26 months after presenting with heart failure, an echocardiogram revealed mild mitral regurgitation with LVEF improvement to 55%. He enjoyed an active lifestyle with regular gym exercise, including cardio and strength training, as well as travel vacations for hiking (Figure 1).

## Discussion

This male patient with a past medical history of RA and NHL was diagnosed with DCM which subsequently improved over the course of a few years. While we could not definitively identify an etiology for the patient’s cardiomyopathy or later improvement in cardiac function, we propose several factors. We considered the possibility that one underlying pathology could be responsible for his reversible DCM, as well as the possibility that there were two separate etiologies: one cause responsible for his decrease in LVEF/LV function and a separate cause responsible for his improvement in LVEF/LV function.

Potential causes of this patient’s reversible DCM with decreased LVEF

One possibility for this patient’s condition is that there was an underlying pathology leading to the development of reversible CM. In developed countries, chronic alcohol abuse is one of the most common causes of adult CM [1]; however, the workup of this patient revealed no evidence of substance use disorder. In addition to alcohol use, acquired CM is commonly caused by infection or medications [1]. For infection-induced CM, viruses are the predominant cause, but bacteria and protozoa can also be possible triggers. Some of the most common viruses associated with CM include adenoviruses, enteroviruses such as coxsackie A/B, and echoviruses [1]. These viruses secrete cardiotropic toxins that are difficult to clear from the heart, leading to cardiomyocyte damage [1]. Parvovirus B19 is another common viral cause of CM but differs from other suspects in that it remains latent in the body [12]. When parvovirus B19 becomes active, its viral proteins mediate the release of damaging pro-inflammatory cytokines, eventually leading to cardiomyocyte apoptosis. Sustained release of pro-inflammatory cytokines triggered by parvovirus B19 can lead to chronic inflammation, and prolonged cardiac inflammation can lead to cardiomyocyte damage and necrosis [12].

While most instances of virus-induced DCM produce irreversible cardiac damage, there are reports suggesting that virus-induced DCM can be reversible. For example, in one case report, a 58-year-old man with a history of coronary artery disease presented with a 10-day history of cough, shortness of breath, and fatigue [13]. Tests revealed influenza H1N1 infection along with cardiac abnormalities, including a reduced LVEF to 30-34% and LV hypokinesis [13]. Treatment with the influenza antiviral oseltamivir was initiated promptly, and the patient’s condition gradually improved. Eight days later, an echocardiogram showed normalization of cardiac function, including an increase in LVEF to 55-59% and normal wall motion [13]. Similarly, in another case report, a 48-year-old woman with advanced cystic fibrosis presented with a two-day history of cough, fever, and worsening shortness of breath [13]. The evaluation revealed an LVEF of less than 20% and severe LV dysfunction. She was treated with medications including furosemide, captopril, digoxin, and intravenous immunoglobulin for her presumed viral infection [13]. A repeat echocardiogram 10 days later showed an LVEF of 65-69% and normal LV function. Additionally, in another case report, a 71-year-old woman with a history of breast cancer presented with respiratory symptoms and cardiovascular issues [14]. She had a complex medical history, including coronary artery disease and mitral regurgitation. Initial admission revealed an inferior wall myocardial infarction and an LVEF of 40%. Over the course of several years, follow-up echocardiograms demonstrated variable improvements in LV function, and five years after her initial presentation, her LVEF improved to 62% [14]. While a viral infection clearly could have been a source of this patient’s reversible CM, the workup revealed no evidence of viral infection or virus-induced CM [14].

The possibility of drug-induced CM was also considered. The patient’s previous and current medication history was evaluated. One of the most common medications associated with drug-induced CM is anthracyclines, including doxorubicin. Anthracyclines are a class of chemotherapeutic medications that have been shown to induce irreversible cardiotoxicity, leading to CM through the development of oxygen-free radicals [5]. These reactive oxygen species create excessive oxidative stress, irreversibly damaging the myocardium via myocyte necrosis and/or apoptosis [5]. While this patient had prior exposure to doxorubicin for treatment of his NHL, one would not expect doxorubicin-induced CM to be reversible.

Similarly, rituximab has been implicated in several publications as a causative factor in DCM [2,4,5]. Some case reports do not directly link rituximab to DCM, but authors believe that rituximab was responsible for a global reduction in LV function [15]. In addition to reports of cardiotoxicity, there are two instances of rituximab-associated, reversible, non-ischemic DCM in the literature (Table 1).

**Table 1:** Reported cases of reversible rituximab-induced dilated cardiomyopathy. PMH: past medical history; DVT: deep vein thrombosis; HTN: hypertension; LBBB: left bundle branch block; LVEF: left ventricular ejection fraction; CLL: chronic lymphocytic leukemia; RBBB: right bundle branch block

Author	Year	Demographics	Previous cardiac history?	Significant PMH	Indication for rituximab infusion	EKG findings; LVEF post-infusion	Onset	LVEF after cessation
Cheungpasitporn et al. [3]	2016	51-year-old male	No	DVT, HTN, dyslipidemia	Membranous nephropathy	LBBB; LVEF = 30%	48 hours after the first infusion	3 months later, LVEF = 52%
Girkar et al. [6]	2022	56-year-old male	No	Not reported	CLL	RBBB; LVEF = 20%	5 days after the first infusion	10 days later, LVEF = 60%; EKG still showed RBBB

These cases primarily occurred during the infusion of the biologic therapy (whereas our patient developed DCM symptoms after treatment with rituximab for seven years), and were generally regarded to be a form of an infusion reaction [2,4]. Rituximab infusion reactions are thought to contribute to both early and late forms of cardiotoxicity; early cardiotoxicity is proposed to arise through cytokine release syndrome and mast cell-mediated reactions and late cardiotoxicity is proposed to arise through serum sickness and tumor lysis mechanisms [5].

This patient was also being treated simultaneously with HCQ, a lysosomotropic agent, which has been occasionally implicated in cases of myopathy. Lysosomotropic agents increase lysosomal pH, which decreases lysosomal activity [16]. This decrease in activity potentially leads to deposition and accumulation of HCQ in cardiomyocyte lysosomes. This proposed lysosomotropic quality of HCQ confers a benefit in the treatment of rheumatic conditions, but it can also be responsible for AV conduction defects and eventual CM [16]. More specifically, BBBs, fascicular blocks, third-degree AV blocks, and changes in the endocardium have been reported in patients receiving HCQ therapy, with reported lysosomal inclusions similar to those observed in Fabry’s disease [16]. HCQ has also been implicated in rare cases of myositis. In one report, a 59-year-old woman who developed RA at 25 years of age was treated with 200 mg of HCQ daily for more than 14 years, along with 25 mg of methotrexate daily [17]. Over the course of those 14 years, she presented to the ED on three separate occasions with heart failure. A cardiac MRI showed bi-ventricular concentric hypertrophy and mild impairment of global LV function [17]. An endomyocardial biopsy was performed due to unexplained LV hypertrophy, which showed vacuoles containing autophagolysosomes [17]. These findings are consistent with what is pathognomonic for HCQ-associated non-dilated CM, and the diagnosis was made as such. Following this diagnosis, the patient’s HCQ was discontinued, and she was treated exclusively with methotrexate [17]. However, the patient’s CM did not improve following cessation of HCQ.

**Figure 2:**
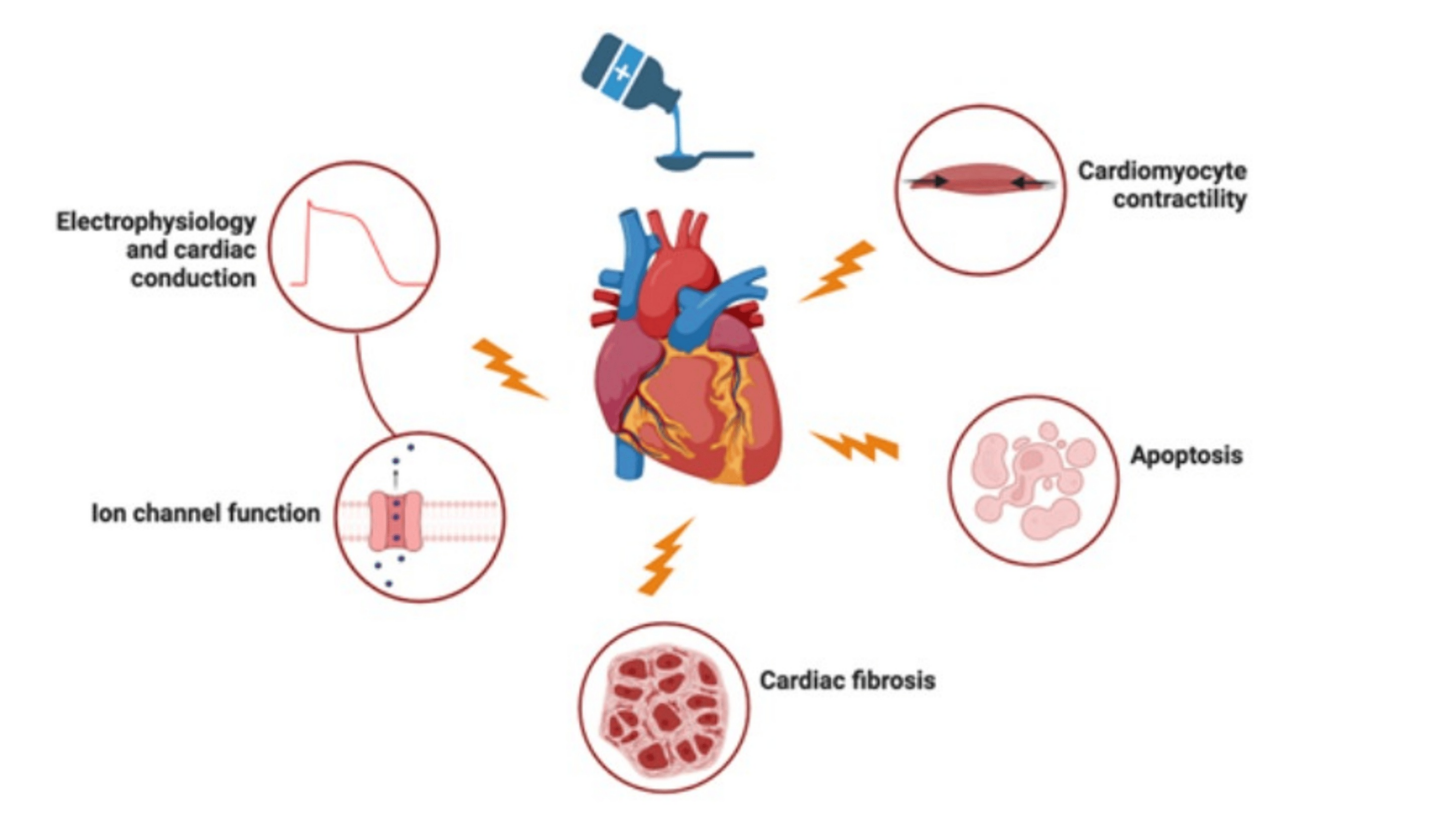
Drug-induced cardiotoxicity. This figure illustrates the possible mechanisms by which medications such as rituximab and hydroxychloroquine may cause cardiotoxicity and result in irreversible cardiac abnormalities. Created using BioRender.com.

In general, drug-induced cardiotoxicity results from the particular way drugs interact with receptors, channels, or proteins within the heart [7]. These interactions can affect enzymatic activities, muscle fiber lengths, and/or vesicle trafficking, ultimately irreversibly altering the mobility/structure of cellular components and disrupting communication pathways [7]. Even upon cessation of the offending agent, cardiac function usually does not return to normal [7]. Overall, rituximab-induced and HCQ-induced DCM appears to be irreversible, with only rare instances where the patient’s DCM reversed; however, in these reversible cases, the onset of DCM occurred during or soon after infusion. Rituximab’s and HCQ’s direct and indirect toxicity on the cardiomyocytes can potentially lead to conduction defects, apoptosis, fibrosis, and stretching of the fibers, all of which are irreversible processes that can affect normal cardiac function [7]. Based on their mechanism of cardiotoxicity, these medications were unlikely to account for this patient’s reversible DCM (Figure 2).

Additionally, there was no concern that rituximab’s and HCQ’s cardiotoxic effects are potentially synergistic or additive with one another. For one, while the pathophysiology of the cardiotoxicity of each drug is understood, it is unclear what the true mechanism is underlying each drug’s individual cardiotoxicity in certain patients but not in others. Additionally, there are no reported cases in the literature suggesting that the administration of both drugs leads to an increased risk of cardiotoxicity. In fact, there are multiple reports concluding that the combination of rituximab and HCQ is a very effective treatment regimen and a standard of care for various inflammatory disorders such as lupus and glomerulonephritis [18,19].

Takotsubo cardiomyopathy, also known as broken-heart syndrome or stress cardiomyopathy, was also included in this patient’s differential. Takotsubo cardiomyopathy is defined as a reversible heart condition that is often triggered by emotional or physical stress [20]. Among the emotional stressors commonly cited are the loss of a loved one, experiences of assault or violence, natural disasters, and significant financial setbacks [20]. Physical stressors that have been documented include sudden illness, surgical procedures, pain, septic conditions, and flare-ups of chronic obstructive pulmonary disease or asthma [20]. Importantly, the absence of an obvious trigger does not preclude diagnosis. In this patient, aside from chronic medical disease (with baseline very good function and lack of disability), we could not identify a specific stressor occurring to the patient.

In Takotsubo cardiomyopathy, there is a sudden weakening of the heart muscle. This can cause symptoms similar to a heart attack such as chest pain, shortness of breath, and arrhythmias [20]. However, unlike a heart attack, there is no blockage in the coronary arteries [20]. The pathophysiology underlying the development of Takotsubo cardiomyopathy is not fully understood, but it is believed that a surge of stress hormones, such as adrenaline, may temporarily stun the heart and impair its ability to pump properly/effectively [20]. While this condition predominantly affects postmenopausal women, it has a reported incidence in men of 10-20% [20].

One defining feature of Takotsubo cardiomyopathy is that it is almost always reversible, and most people recover fully within a few months with supportive care alone (such as medications to manage symptoms and monitoring for complications). However, in rare cases, this condition can lead to more severe complications such as thrombus formation or life-threatening arrhythmias [20]. There are documented cases where individuals present with significant CM and a low LVEF as a result of Takotsubo that reverses over time. In one such case, a 62-year-old female presented with substernal chest pain that radiated to her shoulder blades [21]. Her EKG revealed new T-wave inversions, and her blood work revealed an elevated troponin level, prompting a cardiac catheterization [21]. Left cardiac catheterization showed that she had an LVEF of 35% with LV hypokinesis [21]. She was discharged on supportive therapy; one month later, a repeat echocardiogram showed that her LVEF improved to 55-60% [21].

Potential reasons this patient’s DCM improved with increased LVEF

The significant improvement in cardiac function could potentially be explained either by the withdrawal of rituximab/HCQ or the use of tocilizumab, as this biologic has been documented in the literature to have a positive effect on cardiac function [22]. When the decision was made to stop all DMARDs/biologic therapies, this patient experienced an exacerbation of his previously well-controlled inflammatory RA. Many other potential DMARDs/biologics for the treatment of active RA were relatively contraindicated in this patient due to his history of NHL and ongoing CM. There were a few reasons why the patient was started on IV tocilizumab. First, unlike other biologic agents, tocilizumab has not been reported to increase the risk for lymphoma or CHF [23]. Second and most importantly, there are studies in the literature suggesting that there is no increased risk of coronary heart disease events with the use of tocilizumab. In fact, it is believed that tocilizumab may confer a positive benefit on cardiac function compared to other DMARDs/biologics [22,23]. In a major systematic review conducted, the authors employed a meta-analysis of 14 observational studies for adults with RA treated with tumor necrosis factor inhibitors (TNFis), non-TNFi biologics, and conventional synthetic DMARDs [22]. They found that compared to TNFis, tocilizumab may have been associated with a reduced risk of major adverse cardiovascular events (MACE) [22]. Similarly, in a clinical trial known as “IL-6 Inhibition for Modulating Inflammation After Cardiac Arrest” (IMICA), 80 comatose patients with out-of-hospital cardiac arrests were randomly assigned to a single infusion of tocilizumab versus placebo [23]. Seventy-two hours later, there were observed decreases in leukocyte levels, creatine kinase myocardial band, troponin, and B-type natriuretic peptide [23]. The findings from this IMICA trial provided evidence that tocilizumab could be associated with a reduction of systemic inflammation and myocardial injury [23].

In another pilot study conducted, tocilizumab was prescribed for 52 weeks to female patients with RA who had inadequate clinical response to methotrexate [24]. In this study, subjects with and without RA were recruited from the Itabashi Chuo Medical Center in Japan [24]. Healthy volunteers were recruited for a control group based on age and sex and matched with patients who had moderate-to-severe active RA [24]. All subjects (experimental and control) underwent baseline evaluation of LV function and structure, whereby differences in measurements of LV geometry and function were measured at baseline at the start of the study [24]. Then, measurements between baseline and after 52 weeks of treatment were measured among patients with RA after they were treated with tocilizumab (8 mg/kg IV every four weeks) at the end of the study [24]. The results revealed that tocilizumab significantly increased LVEF, decreased LV mass index, normalized several LV morphological features, and improved the simplified disease index in patients with RA [24]. Although LV geometry in the patients with RA at baseline showed eccentric hypertrophy compared with non-RA controls at the start of the study, this finding also normalized after 52 weeks of tocilizumab treatment in the study [24].

Additionally, more recent studies have further investigated tocilizumab’s cardioprotective effects by assessing its conferred benefit in patients with COVID-19 myocarditis. Results from one study found that tocilizumab demonstrated improvement in patients’ LVEFs within one week of administration, and if given early enough, could completely save a patient’s cardiac function from COVID-19-induced myocarditis [25].

While it is possible that the placement of a biventricular ICD could have been responsible for the improvement of this patient’s symptoms/LVEF, we believe it should have only improved his LVEF very mildly (only about 8%) and should not account for an improvement of nearly 40%. According to the literature, while implantation of a biventricular ICD has been shown to improve symptoms and survival of patients with heart failure, there does not appear to be an associated increase in LVEF [26,27]. In one study conducted by Moss et al. (2002), 1,232 patients with a past medical history of prior LV dysfunction were split into two groups: one group received conventional therapy and the other group received implantable ICD therapy. Each group had similar baseline characteristics (such as low LVEFs averaging around 30%). The results showed that ICD therapy significantly improved survival but did not significantly increase patients’ LVEFs from baseline [27]. Similarly, a meta-analysis of randomized controlled trials and observational studies to assess the efficacy and safety of biventricular ICDs in patients with LV impairment (the primary outcome assessed was the change in LVEF) [26]. After analyzing six randomized controlled trials and 47 observational studies on biventricular pacing, the results showed that biventricular ICDs improved LVEF only by 8.4% [26]. Curiously, there are a few studies in the literature that show biventricular tracing can lead to a higher degree of LVEF recovery in patients with LBBB compared to patients without LBBB [28,29]. While interesting, these findings cannot be taken as definitive, as the patient populations in these studies were small (n = 100 and n = 20, respectively). Despite the studies being underpowered, their findings reveal that the degree of LVEF recovery was 6% [28,29]. This increase of about 6% (improvement of LVEF following ICD placement in patients with LBBB compared to patients without LBBB) on top of the traditionally accepted 8% (effect of ICD placement of LVEF in general) still would not fully account for this patient’s improvement in LVEF of nearly 40%.

Limitations

We understand the limitations of using an echocardiogram alone to measure this patient’s LV function. While LVEF measurements obtained through an echocardiogram are crucial indicators of cardiac function, it is essential to acknowledge the inherent subjectivity and variability in their interpretation. The assessment of LVEF is heavily reliant on the skill and experience of the reader, as well as various technical factors during image acquisition and analysis. For example, this patient’s LVEF increased from 10-15% (Winter 2019) to 50-55% (Spring 2022), which is much greater than the anticipated margin of error. Consequently, discrepancies in LVEF measurements are not uncommon and can lead to significant variations in clinical decision-making. Factors such as image quality, patient positioning, and even subjective judgment calls can introduce considerable error in LVEF calculations. Even considering this shortcoming, we still found it to be an acceptable estimate of this patient’s LV function. At presentation, the patient had acute renal failure related to CHF. For this reason, cardiac catheterization was performed to evaluate coronary arteries alone, and no ventriculogram was completed to limit contrast exposure. We propose that sequential echocardiogram studies performed in the same institution demonstrate the comparative improvement in LV function.

In a similar vein, we do not have access to this patient’s reports from sequential echocardiograms that he would have received while on doxorubicin therapy. Those reports would have provided a comprehensive picture of this patient’s cardiac health trajectory, which would have facilitated better monitoring, treatment optimization, and patient engagement.

## Conclusions

This patient’s long history of medical conditions acted as a physical stressor that led to his reversible DCM and/or decrease in LVEF with global cardiac hypokinesis. Some potential causes include drug-induced (rituximab and/or HCQ), virus-induced, and Takotsubo cardiomyopathy. This patient then experienced an improvement in his symptoms along with an increase in his LVEF back to a normal reference range. While not completely explanatory, two potential causes for this (other than the reversibility of Takotsubo) include the placement of a biventricular ICD and/or tocilizumab infusions. Overall, this case highlights the importance of comprehensive cardiac monitoring in symptomatic patients at high risk for cardiovascular disease, such as this patient with long-standing inflammatory disease. This patient was managed appropriately due to constant interaction between cardiology and rheumatology. Additionally, physicians should continue to closely monitor and consider the serious potential risks of all treatment regimens. It is especially important in patients with multiple cardiovascular risk factors and conduction defects such as prolonged/long QT.
